# Association between ECG parameters and late gadolinium enhancement along the course of myocarditis

**DOI:** 10.1007/s10554-023-02811-3

**Published:** 2023-02-24

**Authors:** Mihály Károlyi, Márton Kolossváry, Lucas Weber, Ioannis Matziris, Malgorzata Polacin, Justyna M. Sokolska, Alexander Gotschy, Hatem Alkadhi, Robert Manka

**Affiliations:** 1 Institute of Diagnostic and Interventional Radiology, University Hospital Zurich, University of Zurich, Zurich, Switzerland; 2 Gottsegen National Cardiovascular Center, Budapest, Hungary; 3 Department of Radiology, Cantonal Hospital Winterthur, Winterthur, Switzerland; 4 Department of Cardiology, University Heart Center, University Hospital Zurich, University of Zurich, Zurich, Switzerland; 5 Institute for Biomedical Engineering, University and ETH Zurich, Zurich, Switzerland; 6 Department of Heart Diseases, Wroclaw Medical University, Wroclaw, Poland; 7 Department of Cardiology, University Heart Center, University Hospital Zurich, Raemistrasse 100, 8091 Zurich, Switzerland

**Keywords:** Electrocardiography, Gadolinium, Magnetic resonance imaging, Myocarditis

## Abstract

Purpose: Numerous electrocardiogram (ECG) abnormalities and late gadolinium enhancement (LGE) in cardiac magnetic resonance imaging (CMR) have been related to poor prognosis in acute myocarditis. We evaluated whether ECG parameters are associated with the distribution and dynamic of LGE along the course of myocarditis. Methods: Fifty-one patients with CMR confirmed acute myocarditis were included who underwent CMR with LGE and 12-lead ECG at baseline and 3-month follow-up at our institution. The association between the presence, regional distribution and change of ECG parameters and LGE was investigated using linear regression analysis. LGE was quantified as visual presence score (VPS) and visual transmurality score (VTS). Results: Among many ECG parameters only > 1 mm ST-elevation (STE) was associated with VPS and VTS at baseline (β = 3.08 [95%CI: 1.75; 4.41], p = < 0.001 and β = 5.40 [95%CI: 1.92; 8.88], p = 0.004; respectively). STE was most frequent in lateral and inferior ECG-leads (48% and 31%) and it was associated with VPS and VTS in these localizations (p < 0.05 all), however no association between anterior-septal STE and LGE could be confirmed. At follow-up the regression of STE was associated with the regression of VPS and VTS in univariate analysis (β=-1.49 [95%CI: -2.41; -0.57], p = 0.003 and β=-4.87 [95%CI: -7.18; -2.56], p = 0.001, respectively), which remained significant for VTS using a multivariate model (β=-2.39 [95%CI: -3.32; -0.47], p = 0.019). Conclusion: Although we demonstrated some promising associations between STE and LGE, the usability of ECG to estimate the territorial involvement and dynamical changes of LGE along the course of myocarditis is generally limited and cardiac magnetic resonance should be considered for this purpose.

## Introduction

Acute myocarditis is an inflammatory disease of the myocardium with recent onset and variable presentation. While most patients only develop flu like symptoms and completely recover, some patients present with a fulminant course and up to 30% may develop left ventricle function impairment with dilatative cardiomyopathy [1]. Early diagnosis and identification of individuals with possible bad clinical outcome is therefore essential to assign appropriate treatment. Invasive endomyocardial biopsy remains the gold standard for diagnosing myocarditis, however, false negative findings related to sampling errors are frequent and complication rates are as high as 8.9% in low-volume centers [2]. Cardiac magnetic resonance (CMR) imaging offers a non-invasive alternative to confirm acute myocyte injury with a high-degree of accuracy and it is the non-invasive gold standard technique for cardiac function assessment, which makes it the ideal test to evaluate patients with potential acute myocarditis. CMR with late gadolinium enhancement (LGE) is also a reliable technique to assess the fibrotic transformation of injured myocardial territories along the temporal course of myocarditis [3, 4]. Moreover, the persistence of LGE after the acute phase of myocarditis, especially in the septal-region has been linked to worst outcome [4]. Hence, patients suffering from acute myocarditis are often assigned to a repeated CMR exam to assess disease persistence and estimate prognosis. Despite of the increasing incidence of acute myocarditis in both US and European registers [5, 6], CMR is still markedly underutilized in many centers in patients with suspected myocarditis, with application rates as low as 29% (range 1–63%) [7]. Therefore, ECG and laboratory tests remain in the front line of the diagnostic work-up and often used in the follow-up of acute myocarditis in many centers [8]. Cardiac biomarkers and inflammatory laboratory parameters were however found recently insufficient to reflect dynamic LGE changes during follow-up in myocarditis, which is well-known predictor of bad outcome [9]. At the same time, until now, there is paucity of data regarding the interrelationship of ECG and CMR pathologies along the course of acute myocarditis. Especially, the value of ECG parameters in the assessment of territorial myocardial injury and the usability of ECG pathologies in the estimation of dynamic of LGE changes along the course of the disease remains unknown. Given this background, we sought to test the hypothesis, that qualitative and quantitative ECG parameters are associated with the presence, regional distribution and dynamics of myocardial injury along the course of acute myocarditis, as compared to CMR.

## Materials and methods

### Study population

Consecutive patients who underwent baseline and follow-up 12-lead ECG and CMR due to suspected acute myocarditis were retrospectively screened within a 4-year time period (January 2016 - December 2019) at our center. Only patients were included, in whom the diagnosis of acute myocarditis could be established in accordance with the respective guidelines [1], and who met the latest Lake Louise Criteria for the CMR diagnosis of acute myocarditis as defined by the Scientific Expert Panel of the American College of Cardiology [10]. Our inclusion criteria were restricted to patients who had baseline ECG and CMR within 7 days from their first clinical episode of suspected acute myocarditis and follow-up at 90 days (+/- 15 days) as part of the routine clinical work-flow at our institution. Coronary artery disease was excluded in all study participants either with invasive coronary angiography, CT coronary angiography or based on low pre-test probability (age: < 30 years old). Patients’ medical records were additionally screened for underlying comorbidities which may potentially affect the myocardium and patients with the following conditions were excluded: former diagnosis of myocarditis, congenital heart disease or prior cardiac surgery, reduced left ventricular ejection fraction or know cardiomyopathies and other systemic condition with potential myocardial involvement, such as collagenosis, eosinophilic disorder, sepsis, tuberculosis with extensive mediastinal or pulmonal manifestation and concurrent malignancy under chemotherapy. According to the strict ethical regulations at our institution written informed consent was retrospectively collected from all study participants and the institutional review board (IRB) approved the study in full conformance with the principles of the Declaration of Helsinki.

### ECG data analysis

12-lead standard ECGs were recorded at 25 mm/s and 10 mm/V according to routine clinical protocol. ECGs at hospital admission (baseline) and at the time of the second CMR (follow-up) were analyzed. Additionally, the change between baseline and follow-up ECGs was evaluated, where “change” was defined the presence of a pathology at baseline and absence at follow-up or vice versa and “no change” was defined as the presence or absence of a pathology both on baseline and follow-up study. Two board certified cardiologists (I.M and M.K.) evaluated ECG recordings in consensus, blinded to clinical and CMR data.

Qualitative and quantitative ECG pathologies with an established association with acute myocarditis were analyzed [11]. Among qualitative EGG parameters, atrial fibrillation, premature ventricular contractions (PVC), fascicular-block (IVB) or high-degree atrioventricular block (AVB), PQ-depression, ST-elevation (STE) or depression (STD) and T-inversion were assessed. >0.5 mm PQ-depression was considered pathological and 1.0 mm was defined as the cut-off value for significant ST-segment changes, as assessed 60ms after the J-point.[12] Regional distribution of these ECG changes was additionally evaluated, considering V1-V4 and aVR leads as anterior-septal; II, III and aVF leads as inferior; and I, aVL, V5-V6 leads as lateral regions, respectively. Quantitative ECG parameters were collected from the automated recordings of the ECG-registers and were validated by the observers. Among quantitative parameters pathological QRS-T angle (> 100^o^), wide QRS complex (> 120 ms) and prolonged QT or QTc interval (> 420 ms) were evaluated. QRS-T angle was defined as the absolute difference between the frontal plane QRS and T axis values and it was adjusted for an angle > 180^o^ (360^o^ – angle) [13].

### CMR imaging protocol and data analysis

Studies were either performed on a 1.5 Tesla (Achieva, Philips Medical Systems, Best, The Netherlands) or 3.0 Tesla (Skyra, Siemens, Erlangen, Germany) magnetic resonance scanner with dedicated setup for cardiac examinations. Cine sequences were acquired with retrospective ECG-triggering under expiration in standard orientations (2-chamber, 3-chamber, 4-chamber view and short axis). Native T1 and T2 Mapping, as well as T2-weighted images sequences of the left ventricle (LV) were obtained in one to three short-axis slices (base, mid-ventricular and apex) to assess myocardial edema. LGE images were acquired 10-minutes after gadolinium administration in standard orientations (short axis stack from base to apex, 2, 3 and 4 chamber view). The optimal inversion time was determined using an inversion time scout sequence.

**Fig. 1:**
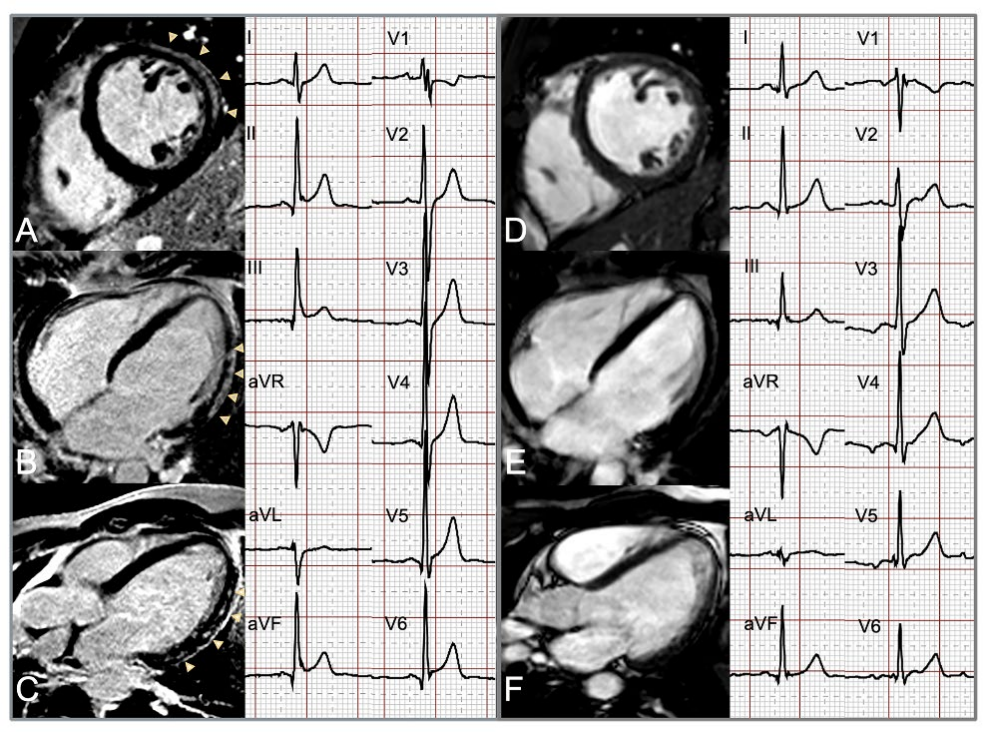
Example for change of ST-elevation on ECG and late-gadolinium enhancement on CMR from hospital admission to 3-month follow-up in a 31-year-old male patient suffering from acute myocarditis. The left panel shows the baseline CMR images in the SA (A), 4CH (B) and 3CH (C) orientations with extensive subepicardial LGE in the antero-/inferolateral (basal to apical) myocardial segments (marked with arrows) and corresponsive ECG at the time of the baseline CMR with > 1 mm ST-elevation in the II, III, aVF, V5-V6 leads, matching to the infero-lateral myocardial regions. The right panel demonstrates the complete regression of LGE on the follow-up CMR examination, as seen on SA (D), 4CH (E) and 3CH (F) views, with resolving ST-pathologies on concomitant ECG. SA: short-axis; 4CH: four-chamber; 3CH: three-chamber

The CMR diagnosis of myocarditis was established if regional or global myocardial edema was present (defined as an increase of native T2-signal or T2-mapping value) and concomitant signs of a myocardial injury were existing (expressed as increased native T1-mapping value, elevated extracellular volume or positive LGE) [10]. LGE involvement of the LV was further evaluated using a commercial post-processing software (Intellispace Portal. Version 8. Philips Healthcare). Extent of LGE was assessed visually according to the 17- segment model of the American Heart Association (AHA) [14]. For each myocardial segment of the LV the presence and transmurality (0 = no LGE, 1 = < 25%, 2 = 26–50%, 3 = 51–75%, 4 = 76–100%) of LGE was determined and was further combined into a visual presence score (VPS) and visual transmurality score (VTS), as multiplied with the number of affected myocardial segments [15]. The resultant VPS and VTS ranged between 0–17 and 0–68, respectively, and were considered as the representative measures of the global LV involvement of myocardial injury. Regional distribution of LGE was further evaluated in concordance with the 17-segment model of the AHA, where myocardial segments 1,2,7,8,13,14,17 represent the anterior-septal, 3,4,9,10,15 the inferior and 5,6,11,12,16 the lateral regions, respectively [14]. Additionally, CMR findings compatible with associated pericarditis were recorded, defined as pericardial enhancement in the LGE sequences and/or associated pericardial effusion. CMR studies at baseline and at follow-up were evaluated in consensus among two readers experienced in cardiovascular imaging (M.K. 12 years and J.S. 5 years), blinded to clinical and ECG data.An example for baseline and follow-up ECG and CMR in a patient with acute myocarditis is shown in Figure 1.

### Statistical analysis

We used the Shapiro-Wilk-test to assess the normality of continuous variables. Continuous variables are reported as mean ± standard deviation or median and interquartile range (IQR). Categorical variables are expressed as numbers and percentages. Data analysis was performed on a patient and regional (anterior-septal, inferior and lateral) level. According to the distribution of continuous variables, paired T-test and Wilcoxon signed rank test were used to compare patient characteristics, and the McNemar Test and Wilcoxon signed rank test were used to compare ECG and VPS/VTS between baseline and follow-up studies, respectively. The regional distribution of LGE was compared with Friedman’s ANOVA test. To assess the associations between ECG and LGE parameters, we used univariate linear and logistic regression analysis. In case any of the predictors was significant at a p < 0.05 level in univariate analysis, we included it into a multivariate model with all other significant predictors. A P-value < 0.05 was considered to indicate statistical significance. All analyses were performed using commercially available software (SPSS, version 23; Armonk, NY and R, version 3.6.1; https://www.r-project.org).

## Results

### Baseline characteristics

51 patients met our selection criteria and were included in the study (mean age 32 ± 13 years, 9 female). Patients’ baseline characteristics are summarized in Table 1.

**Table 1:** Patient characteristics

	n = 51	
**Baseline parameter**		
Age (years)	32 ± 13	
Male	42 (82)	
BMI (kg/m^2^)	27 ± 5	
**Cardiovascular risk factors**		
Hypertension	5 (10)	
Diabetes	2 (4)	
Dyslipidemia	3 (6)	
Smoking	24 (47)	
active smoker	18 (35)	
Family risk of CAD	12 (24)	
**Clinical presentation**		
Chest pain	44 (86)	
Dyspnea	10 (20)	
Palpitation	9 (18)	
Dizziness or syncope	5 (10)	
Fever	15 (29)	
Recent respiratory infection or flu	22 (43)	
Recent gastrointestinal infection	14 (27)	
Recent other infection	4 (6)	
**Laboratory parameter at admission**		
CRP (mg/ml)	26 (IQR: 7–66)	
CK (U/l)	218 (IQR: 109–426)	
Hs-TnT (ng/l)	303 (IQR: 134–803)	
Myoglobin (µg/l)	33 (IQR: 23–75)	
NT-proBNP (ng/l)	177 (IQR: 71–734)	
**Timing of examinations**		
Admission to baseline CMR (days)	2 (IQR: 1–3)	
Baseline to follow-up CMR (days)	93 (81–110)	
Follow-up ECG and CMR (days)	8 (1–11)	

Data as mean ± standard deviation or count and percentage (%).

Abbreviations: CRP = C-reactive protein, CK = creatine kinase, Hs-TnT =

high sensitive troponine, NT-proBNP = N-terminal pro B-type natriuretic

peptide

### ECG findings at baseline and at follow-up

86% of the patients had an ECG abnormality at hospital admission, which decreased to 56% at follow-up (p < 0.001). Among qualitative ECG abnormalities, ST-T changes (> 1 mm STE or STD and T inversion) were most frequently documented at admission, overall, in 55% of the patients and persisted in 29% at follow-up (p = 0.007). Among quantitative ECG changes, pathological QTc interval was most frequently observed at baseline, overall, in 37% of the patients, which decreased to 8% at follow-up (p = 0.007). ECG findings at baseline and at follow-up are summarized in Table 2. When considering the regional distribution of the qualitative ECG findings, PQ depression was found exclusively in the inferior and lateral ECG leads (50% and 50%, respectively). Among ST-T changes, STD was found in the anterior-septal and lateral regions (75% and 25%, respectively), STE was mostly observed in the lateral and less frequent in the inferior and anterior-septal regions (48%, 31% and 21%, respectively) and T-inversion was most common in the inferior and less frequent in the lateral and anterior-septal regions (74%, 17%, and 9%, respectively). None of the myocardial territories which deemed “healthy” at baseline ECG showed new ECG abnormalities at follow-up.

**Table 2:** ECG and CMR diagnostic parameters at baseline and at 3-month follow-up

n = 51			
**ECG parameters**	***Baseline***	***Follow-up***	***p-value***
Non-sinus rhythm	1 (2)	1 (2)	1.000
Ventricular extrasystole	3 (6)	2 (4)	1.000
Atrioventricular-block	1 (2)	1 (2)	1.000
Fascicular-block	2 (4)	5 (10)	0.250
> 0.5 mm PQ depression	2 (4)	1 (2)	1.000
> 1 mm ST-segment elevation	16 (31)	1 (2)	< 0.001
> 1 mm ST-segment depression	3 (6)	1 (2)	0.500
T-inversion	19 (37)	14 (27)	0.332
Pathological QRS-T angle (> 100^o^)	4 (8)	2 (4)	0.500
Wide QRS complex (> 120 ms)	2 (4)	0 (0)	1.000
Prolonged QTc interval (> 420 ms)	19 (37)	8 (16)	0.007
**CMR parameters**	***Baseline***	***Follow-up***	***p-value***
LVEDDi (mm/m^2^)	27 ± 3	27 ± 3	0.231
Septum thickness (mm)	8 ± 1	8 ± 1	0.702
Lateral wall thickness (mm)	9 ± 2	8 ± 1	< 0.001
LVEF (%)	57 ± 7	57 ± 5	0.619
LVEDVi (ml/m^2^)	84 ± 16	82 ± 16	0.199
LVESVi (ml/m^2^)	37 ± 11	35 ± 9	0.273
LV mass index (g/m^2^)	60 ± 14	51 ± 9	< 0.001
RVEF (%)	59 ± 10	60 ± 6	0.852
RVEDVi (ml/m^2^)	79 ± 15	80 ± 17	0.390
RVESVi (ml/m^2^)	33 ± 8	32 ± 10	0.285
Visual presence score (VPS)	3 (IQR: 1–5)	2 (IQR: 1–4)	< 0.001
Visual transmurality score (VTS)	6 (IQR: 3–11)	3 (IQR: 1–6)	< 0.001

Data as mean ± standard deviation, median and interquartile range (IQR) or count and percentage (%), as appropriate. Abbreviations: LVEDDi = left ventricular end diastolic diameter index, LVEF = left ventricular ejection fraction, LVEDVi = left ventricular end diastolic volume index, LVESVi = left ventricular end systolic volume index, RVEF = right ventricular ejection fraction, RVEDVi = right ventricular end diastolic volume index, RVESVi = right ventricular end systolic volume index

### CMR findings at baseline and at follow-up

96% of the patients showed LGE in a non-ischemic (subepicardial) localization in at least one myocardial segment. 19% of the patients had CMR findings comparable with associated pericarditis. LGE was most frequent in the lateral, and less common in inferior and anterior myocardial regions (90%, 53% and 37%, respectively, p < 0.001). 45% of the LGE positive patients at baseline showed a regression of VPS and 82% of the patients a regression of VTS at follow-up CMR. None of the patients showed an increase of VTS/VPS on follow-up CMR. LGE, as well as functional CMR data at baseline and at follow-up are reported in Table 2. Notably, none of the patients demonstrated persistent myocardial edema on follow-up. Furthermore, a significant decrease in the lateral LV wall thickness and LV mass index was observed from baseline to follow CMR, which also well reflects the decrease of myocardial edema along the course of myocarditis.

### Association between ECG and CMR abnormalities

Among baseline qualitative ECG parameters > 1 mm STE showed an association with baseline VPS and VTS. The presence of > 1 mm STE at baseline ECG was associated with 3.1x increase of VPS (β = 3.08 [95%CI: 1.75; 4.41], p < 0.001) and 5.4x increase of VTS on baseline CMR (β = 5.40 [95%CI: 1.92; 8.88], p = 0.004) in multivariate models. No association between baseline quantitative ECG parameters and baseline LGE was found. The associations between baseline ECG parameters and LGE is summarized in Table 3.

**Table 3:** Association between ECG parameters and semi-quantitative LGE CMR scores at baseline

Outcome	Predictor	Univariate analysis				Multivariate analysis			
		Beta	95% CI		p-value	Beta	95% CI		p-value
VPS	Non-sinus rhythm	-3.58	-8.8	1.64	0.19	-	-	-	-
	Ventricular extrasystole	-1.6	-4.7	1.49	0.32	-	-	-	-
	Atrioventricular-block	0.5	-4.81	5.81	0.85	-	-	-	-
	Fascicular-block	-3.13	-6.82	0.56	0.85	-	-	-	-
	> 0.5 mm PQ depression	-1.57	-5.34	2.2	0.42	-	-	-	-
	> 1 mm ST- elevation	3.08	1.75	4.41	**< 0.001**	3.08	1.75	4.41	**< 0.001**
	> 1 mm ST- depression	1.94	-1.15	5.02	0.22	-	-	-	-
	T-inversion	-0.56	-2.08	0.95	0.47	-	-	-	-
	Pathological QRS-T angle	-0.02	-0.04	0.01	0.18	-	-	-	-
	Wide QRS complex	0.02	-0.02	0.06	0.34	-	-	-	-
	Prolonged QTc interval	-0.02	-0.04	< 0.01	0.05	-	-	-	-
VTS	Non-sinus rhythm	-7.78	-20.15	4.59	0.19	-	-	-	-
	Ventricular extrasystole	-3.15	10.5	4.21	0.41	-	-	-	-
	Atrioventricular-block	0.38	-12.18	12.94	0.95	-	-	-	-
	Fascicular-block	-6.32	-15.17	2.42	0.16	-	-	-	-
	> 0.5 mm PQ depression	-2.74	-11.68	6.21	0.55	-	-	-	-
	> 1 mm ST- elevation	6.46	3.17	9.75	**< 0.001**	5.4	1.92	8.88	**0.004**
	> 1 mm ST- depression	9.6	2.71	16.5	**0.008**	5.67	-1.2	12.54	0.11
	T-inversion	-1.59	-5.16	1.99	0.39	-	-	-	-
	Pathological QRS-T angle	-0.05	-0.1	0.003	0.069	-	-	-	-
	Wide QRS complex	0.08	-0.01	0.16	0.09	-	-	-	-
	Prolonged QTc interval	-0.03	-0.08	0.02	0.27	-	-	-	-

All parameters significant in univariate analysis (p < 0.05) were included in multivariate models

When assessing the regional interrelationship between ECG parameters and LGE, > 1 mm STE at baseline showed a regional association with baseline VPS and VTS in the inferior and lateral localizations. No association in the anterior-septal localization between ECG and CMR pathologies could be revealed. The regional associations between baseline ECG parameters and LGE is summarized in Table 4. When considering the changes of ECG and LGE parameters between baseline and follow-up, among patients who showed > 1 mm STE at baseline, the regression of STE at follow-up ECG was associated with the regression of both VPS and VTS at follow-up in univariate analysis (β=-1.49 [95%CI: -2.41; -0.57], p = 0.003 and β=-4.87 [95%CI: -7.18; -2.56], p = 0.001, respectively), which remained significant for VTS using a multivariate model (β=-2.39 [95%CI: -3.32; -0.47], p = 0.019). The associations between the change of ECG abnormalities between baseline and follow-up ECG and LGE between baseline and follow-up CMR is summarized in Table 5.

**Table 4:** Association between regional ST-changes on ECG with regional LGE values on CMR at baseline

*Region*	Outcome	Predictor	Univariate analysis				Multivariate analysis			
			**β**	**95% CI**		**p-value**	**β**	**95% CI**		**p-value**
*Anterior-septal*	VPS	> 1 mm ST- elevation	0.11	-0.79	1.00	0.81	-	-	-	-
		> 1 mm ST- depression	0.10	-1.13	1.33	0.87	-	-	-	-
		T-inversion	0.45	-1.04	1.94	0.56	-	-	-	-
	VTS	> 1 mm ST- elevation	-0.13	-1.87	1.60	0.88	-	-	-	-
		> 1 mm ST- depression	-1.06	-1.06	3.64	0.29	-	-	-	-
		T-inversion	0.92	-1.95	3.78	0.53	-	-	-	-
*Lateral*	VPS	> 1 mm ST- elevation	1.60	0.74	2.46	**< 0.001**	1.60	0.74	2.46	**< 0.001**
		> 1 mm ST- depression	0.82	-2.30	3.94	0.61	-	-	-	-
		T-inversion	0.33	-1.28	1.94	0.69	-	-	-	-
	VTS	> 1 mm ST- elevation	3.46	1.06	5.86	**0.007**	3.46	1.06	5.86	**0.007**
		> 1 mm ST- depression	7.44	-0.63	15.51	0.08	-	-	-	-
		T-inversion	-1.04	-5.33	3.25	0.64	-	-	-	-
*Inferior*	VPS	> 1 mm ST- elevation	0.85	0.28	1.42	**0.005**	0.79	0.24	1.35	**0.008**
		> 1 mm ST- depression	-	-	-	-	-	-	-	-
		T-inversion	-0.50	-0.98	-0.02	**0.045**	-0.43	-0.88	0.02	0.07
	VTS	> 1 mm ST- elevation	1.72	0.18	3.26	**0.033**	1.72	0.18	3.26	**0.033**
		> 1 mm ST- depression	2.29	-0.82	5.39	0.16	-	-	-	-
		T-inversion	-0.94	-2.22	0.34	0.16	-	-	-	-

In the territorial analysis we compared ST-changes on ECG with LGE on a regional basis, in the anterior-septal, lateral and inferior regions

All parameters significant in univariate analysis (p < 0.05) were included in multivariate models

**Table 5:** Association between changes in ECG parameters and semi-quantitative LGE CMR scores from baseline to follow-up

ECG parameter	Baseline	Follow-up	*Univariate analysis*								*Multivariate analysis*								
			VPS change				VTS change				VPS change					VTS change			
			*β*	*95%CI*		*p value*	*β*	*95% CI*		*p value*	*β*	*95%CI*			*p value*	*β*	*95%CI*		*p value*
NS rhythm	-	+	1.12	-2.07	4.32	0.50	3.78	-4.76	12.31	0.39	**-**	**-**	-	**-**		**-**	**-**	**-**	**-**
	+	-	1.12	-2.08	4.32	0.50	3.78	-4.76	12.31	0.39	**-**	**-**	**-**	**-**		**-**	**-**	**-**	**-**
	+	+	**-**	**-**	**-**	**-**	**-**	**-**	**-**	**-**	**-**	**-**	**-**	**-**		**-**	**-**	**-**	**-**
VES	-	+	1.15	-2.07	4.37	0.49	3.81	-4.84	12.46	0.39	**-**	**-**	**-**	**-**		**-**	**-**	**-**	**-**
	+	-	1.15	-1.15	3.45	0.33	1.31	-4.87	7.49	0.07	**-**	**-**	**-**	**-**		**-**	**-**	**-**	**-**
	+	+	0.15	-3.07	3.37	0.93	2.81	-5.84	11.46	0.53	**-**	**-**	**-**	**-**		**-**	**-**	**-**	**-**
AV-block	-	+	**-**	**-**	**-**	**-**	**-**	**-**	**-**	**-**	**-**	**-**	**-**	**-**		**-**	**-**	**-**	**-**
	+	-	**-**	**-**	**-**	**-**	**-**	**-**	**-**	**-**	**-**	**-**	**-**	**-**		**-**	**-**	**-**	**-**
	+	+	0.08	-3.16	3.26	0.96	-0.38	-8.96	8.20	0.93	**-**	**-**	**-**	**-**		**-**	**-**	**-**	**-**
FA-block	-	+	-0.58	-2.46	1.30	0.59	-8.78	-13.19	-4.38	**< 0.001**	-	-	-	**-**		-2.70	-6.29	0.86	0.15
	+	-	**-**	**-**	**-**	**-**	**-**	**-**	**-**	**-**	**-**	**-**	**-**	**-**		**-**	**-**	**-**	**-**
	+	+	1.09	-1.19	3.36	0.35	2.72	-2.62	8.06	0.323	**-**	**-**	**-**	**-**		0.09	-3.66	3.83	0.96
PQ depression	-	+									**-**	**-**	**-**	**-**		-	-	-	-
	+	-	1.08	-2.12	4.29	0.51	-1.37	-10.01	7.28	0.76	**-**	**-**	**-**	**-**		**-**	**-**	**-**	**-**
	+	+	0.91	-4.12	2.29	0.58	1.63	-7.01	10.28	0.71	**-**	**-**	**-**	**-**		**-**	**-**	**-**	**-**
ST- elevation	-	+	**-**	**-**	**-**	**-**	**-**	**-**	**-**	**-**									
	+	-	-1.49	-2.41	-0.57	**0.003**	-4.87	-7.18	-2.56	**0.001**	-0.47	-1.43	0.49	0.34		-2.39	-4.32	-0.47	**0.02**
	+	+	-0.34	-2.46	1.77	0.75	1.34	-3.97	6.66	0.62	0.44	-1.46	2.35	0.65		2.67	-1.07	6.34	0.17
ST- depression	-	+	**-**	**-**	**-**	**-**	**-**	**-**	**-**	**-**									
	+	-	-0.94	-3.22	1.34	0.42	-7.08	-12.90	-1.27	**0.021**	**-**	**-**	**-**	**-**		-0.63	-4.76	3.50	0.77
	+	+	1.06	-2.13	4.26	0.52	3.42	-4.72	11.55	0.41	**-**	**-**	**-**	**-**		10.57	5.08	16.06	**< 0.001**
T-inversion	-	+	-0.01	-1.46	1.44	0.99	0.83	-3.07	4.74	0.68	**-**	**-**	**-**	**-**		**-**	**-**	**-**	**-**
	+	-	-0.03	-1.18	1.13	0.96	0.36	-2.74	3.46	0.82	**-**	**-**	**-**	**-**		**-**	**-**	**-**	**-**
	+	+	0.53	-0.77	1.83	0.43	1.25	-2.24	4.74	0.49	**-**	**-**	**-**	**-**		**-**	**-**	**-**	**-**
QRS-T angle change [°]			0.008	-0.004	0.021	0.20	0.023	-0.011	0.057	0.19	**-**	**-**	**-**	**-**		**-**	**-**	**-**	**-**
QRS interval change [msec]			0.001	-0.009	0.011	0.79	-0.006	-0.033	0.021	0.67	**-**	**-**	**-**	**-**		**-**	**-**	**-**	**-**
QTc change [msec]			-0.003	-0.017	0.011	0.70	-0.011	-0.048	0.027	0.58	**-**	**-**	**-**	**-**		**-**	**-**	**-**	**-**

Multivariate models were corrected for baseline VPS and VTS values as their values may affect overall change

+ and - signs represent the presence of the absence of the given abnormality at baseline or follow-up

## Discussion

Our study demonstrated that among several frequently seen ECG abnormalities in acute myocarditis, only > 1 mm ST-elevation correlates with the presence and extent of LGE on CMR. STE was most frequent in the lateral and inferior myocardial territories in our cohort and though we showed a regional interrelationship of STE with LGE in these regions, no association between STE and LGE in the anterior-septal territories could be demonstrated. Importantly, the regression of STE from baseline to follow-up ECG was associated with the decrease of LGE at 3-months’s follow-up CMR, while no other ECG parameters showed a distinct association with LGE.

Acute myocarditis carries varying outcome and CMR deems extremely helpful in the prediction of a long-term prognosis. A meta-analysis found that positive LGE in myocarditis is a strong predictor of future adverse events (OR 5.85%; 95%CI: 2.88; 11.86) [16], and a recent multi-center registry showed that persistent LGE without myocardial edema on repeated CMR at 6-months is associated with worse outcome along a 7-years clinical follow-up [4]. Additionally, the extension and localization of LGE is also important in cardiovascular risk prediction, as extensive LGE (involving > 2 myocardial segments) and antero-septal localization carry independent prognostic risk for adverse outcome in myocarditis [17]. Notably, LGE completely resolves in 10% of the patients, which suggests that LGE in the acute phase of myocarditis does not mean definite fibrosis [4]. Therefore, a repeated estimation of risk for future cardiovascular events in myocarditis seems beneficial with CMR, however, its application strongly depends on local availability and expertise. Additionally, due to age related comorbidities and reduced compliance younger individuals are more likely to undergo CMR in suspected myocarditis than older patients [7].

If CMR is not available, estimation of prognosis, moreover, risk-monitoring with conventional diagnostic techniques only is challenging. ECG pathologies in the acute phase of myocarditis are frequent, yet, not specific. Still, certain baseline ECG abnormalities have been linked to the development of myocardial fibrosis and worse outcome in myocarditis [11]. Furthermore, the persistence of repolarization abnormalities are often considered as markers of a definitive fibrosis and inferior outcome, while the regression of ECG abnormalities at follow-up are regularly interpreted as a resolving disease [18]. Accordingly, several ECG parameters have been tested to tailor patient management and estimate long-term prognosis with variant results. In a cohort of 388 patients with MINOCA (myocardial infarction with non-obstructed coronary arteries), STE at admission was found the best ECG predictor of mortality in a median follow-up of 3.5 years [19]. Hausvater et al. found also an association of STE in myocarditis with elevated risk of long-term major adverse cardiac events (MACE) as well as with early mortality, however, they showed no correlation between STE and CMR findings [20]. At the same time Chen et al. found no association between STE and a fulminant course of acute myocarditis [21].

In our study we found that ST-pathologies are the most common ECG findings related to acute myocarditis. >1 mm STE at baseline was associated with a 3.1-fold increase of VPS and 5.4-fold increase of VPS on baseline CMR. STE was most frequently related to lateral and inferior myocardial segments (48% and 31%). We could demonstrate a regional interrelationship between STE and LGE localized to lateral and inferior myocardial regions. While an earlier study could not demonstrate an association between STE and myocardial damage in CMR [22], similar to similar to our findings, Deluigi et al., who compared lateral and septal myocardial segments in patients with acute myocarditis, found also a correlation of STE with LGE between these regions [23]. At the same time, we could not demonstrate an association between STE and LGE in the anterior-septal localizations, which is an important finding, considering that septal LGE in myocarditis was previously found to carry an elevated risk for MACE (HR 2.55), as compared to other myocardial regions (HR 1.37–1.82) [24]. Notably, the number of patients with septal ECG pathologies and septal LGE were lowest in our cohort, which might limit our findings. Importantly, we found an association between the regression of STE and the regression of VPS and VTS at 3-month, which result emphasizes the value of evaluating the change STE along the course of myocarditis. Notably, among other qualitative ECG parameters, STD also showed an association with VTS at baseline in univariate analysis, which however did not reach the level of significance in multivariate analysis and no clear association between the change of STD and LGE along the follow-up could be revealed.

Among quantitative ECG parameters, wide QRS-T angle (≥ 100^o^) was previously found to strongly correlate with the presence of LGE in CMR [25]. Additionally, pathologic QRS-T angle was previously linked to elevated risk of mortality and heart failure in acute myocarditis [13]. Contrary to these, our results did not show an association between pathological QRS-T angle or any other quantitative ECG parameters and LGE. More importantly, none of the assessed quantitative ECG parameters deemed useful to monitor the change of LGE along a 3-month follow-up. Our findings need to be validated in larger studies.

Our study has more limitations. First, our study is limited by the retrospective analysis with the lack endomyocardial biopsy as reference standard and the lack of a long-term clinical follow-up. Nevertheless, large multi-center studies have already addressed the long-term predictive value of ECG and CMR parameters in myocarditis [6, 13, 17], and this was not the focus of our study. Second, our sample size was only moderate (n = 51), however, we used strict study selection criteria to avoid any preexisting comorbidity that could have altered our findings. Third, we did evaluate the association between myocardial edema and ECG pathologies, as per standard protocol T2 and T2-mapping datasets were acquired only in one to three short-axis orientations and deemed insufficient for a detailed regional analysis of the whole left ventricular myocardium. Notably, contrary to LGE, myocardial edema usually dissolves after the acute phase of myocarditis, as it was also completely resolved in all patients of our cohort. Furthermore, a previous study showed no association between persistent myocardial edema and bad prognosis, the presence myocardial edema on follow-up was rather suggestive of a residual chance of recovery [4]. Fourth, we choose to assess LGE using a visual scoring system, instead of a more precise quantification with a semiautomated technique, e.g., the full width at half maximum (FWHM) method. The reason behind this is that we aimed to use the most reproducible method for LGE quantification in our follow-up study. As myocarditis mostly affects the subepicardial region, even a slight difference in the epicardial contouring might significantly impact intra- and inter-observer reproducibility of semi-automated techniques if the delineation of epicardial LGE from epicardial fat is false. Additionally, the quantitative estimation of LGE is also highly dependent on the used thresholds. In accordance, the higher reproducibility of visual scores over semi-automated techniques for LGE quantification in myocarditis was demonstrated in multiple studies [15, 26]. Moreover, visual LGE scores were previously proven to have similar association with MACE in myocarditis as quantitatively assessed LGE [15].

## Conclusion

In patients with suspected myocarditis among several qualitative and quantitative ECG parameters only ST elevation is associated with LGE on CMR, which may be used as a gatekeeper to identify individualist at risk of myocardial injury and future cardiac events. If CMR is not available, monitoring the resolution of ST elevation at follow-up seems to be valuable to estimate the dynamic of LGE, which is a well-known marker of fibrotic transformation of injured myocardium. While we showed some promising results regarding the territorial associations between STE and LGE in the inferior and lateral localizations as well, no association in the anterior-septal region could be revealed. This overall suggests, that the capability of ECG is limited to estimate the myocardial involvement along the course in myocarditis and CMR should be considered for this purpose.

## Data Availability

The datasets used and/or analyzed during the current study are available from the corresponding author on reasonable request.

## Abbreviations

AHA: American Heart Association
CAD: Coronary artery disease
CMR: Cardiac magnetic resonance
ECG: Electrocardiogram
ESC: European Society of Cardiology
LGE: Late gadolinium enhancement
LV: Left ventricle
MACE: Major adverse cardiac events
MINOCA: Myocardial infarction with non-obstructed coronary arteries
STD: >1 mm ST-segment depression
STE: >1 mm ST-segment elevation
T: Tesla
VPS: Visual presence score
VTS: Visual transmurality score
